# Point Cloud Based Relative Pose Estimation of a Satellite in Close Range

**DOI:** 10.3390/s16060824

**Published:** 2016-06-04

**Authors:** Lujiang Liu, Gaopeng Zhao, Yuming Bo

**Affiliations:** School of Automation, Nanjing University of Science and Technology, Nanjing 210094, China; liulujiang@njust.edu.cn (L.L.); byming@njust.edu.cn (Y.B.)

**Keywords:** pose estimation, point cloud, pose initial acquisition, pose tracking

## Abstract

Determination of the relative pose of satellites is essential in space rendezvous operations and on-orbit servicing missions. The key problems are the adoption of suitable sensor on board of a chaser and efficient techniques for pose estimation. This paper aims to estimate the pose of a target satellite in close range on the basis of its known model by using point cloud data generated by a flash LIDAR sensor. A novel model based pose estimation method is proposed; it includes a fast and reliable pose initial acquisition method based on global optimal searching by processing the dense point cloud data directly, and a pose tracking method based on Iterative Closest Point algorithm. Also, a simulation system is presented in this paper in order to evaluate the performance of the sensor and generate simulated sensor point cloud data. It also provides truth pose of the test target so that the pose estimation error can be quantified. To investigate the effectiveness of the proposed approach and achievable pose accuracy, numerical simulation experiments are performed; results demonstrate algorithm capability of operating with point cloud directly and large pose variations. Also, a field testing experiment is conducted and results show that the proposed method is effective.

## 1. Introduction

Relative navigation is a key functionality for emerging new mission needs in automated rendezvous and docking, active debris removal, on-orbit servicing missions, *etc.* [1,2,3,4]. It is essential for collision avoidance. Up to now, several technology demonstration missions are designed for cooperative targets, which have aids such as markers optical reflectors [5,6,7]. It is still an open research area facing many technical challenges for uncooperative targets. One of the greatest challenges is to acquire the relative pose *i.e.*, the six degrees-of-freedom (6DOF) pose, between the target and the chaser which are, in general, considered to be moving independently.

Pose estimation is the process of estimating relative attitude and position, which depends on largely on the type of sensor. Usually stereo vision [6,8,9,10], monocular vision [11,12,13] and LIDAR (light detection and ranging) [4,7,14,15,16,17,18,19] sensor are the sensor types used in space applications. Stereo vision approaches provide measurements of high accuracy at a high rate, but their working distance is limited to the baseline and the computational requirements increase with the increase of resolution. Monocular vision lacks of depth information, it often needs other additional information. LIDAR sensors, which output is the point cloud data, are robust against lighting changes. Each point cloud data contains a range vector in the sensor frame. In recent years, flash LIDAR [4,7,15,16,17,18] has discussed for pose estimation. Different from scanning LIDAR which employs a scanning device to collect one point cloud data at a time, flash LIDAR can collect the entire point cloud data image at once. This characteristic helps reduce distortion in the point cloud data and provide pose estimation result at a fast frame rate. However, the drawbacks are also obvious, such as low resolution (*i.e.*, the typical resolution is less than 256 × 256) [4], and measurement noise *et al.*

The relative navigation in close range is still an open research area; especially the target satellite has no cooperative markers. This paper focuses on the process of 6DOF pose estimation in close range for satellite using the flash LIDAR sensor. Assuming that the 3D model of the target is known, a novel relative pose estimation method is proposed by directly aligning the sensor point cloud data and the model point cloud data. Different from the existing works, this method has no need for the process of feature detection and feature tracking; it directly aligns the dense point cloud data to realize the pose initial acquisition and the pose tracking. A simulation system is proposed to generate the simulated point cloud data based on the model point cloud data which can be used to simulate various conditions for actual motion and evaluate the performance of the sensor and pose algorithm.

The paper is organized as follows: In Section 2, the related works in recent years are described in detail. Details of the proposed pose estimation method are presented in Section 3. In Section 4, the simulation system is introduced in detail. Some experimental results are shown and discussed in Section 5. Finally the work is concluded in Section 6 with a discussion of the limitations and future works.

## 2. Related Work

Relative pose estimation is an important process in the relative navigation of satellites in space. Six degrees-of-freedom pose estimation of relative motion is the key problem. The chaser is typically equipped with sensors that collect the images or point cloud data of the target to estimate the pose.

Many studies have been conducted in recent years. The relative navigation sensor is often designed and tested according to the specific space tasks. Optical vision sensor is adapted to obtain the relative attitude and position in the close approach phase. Stereo vision is most frequently used sensor [6,8,9,10]. The Argon system [6] is the typical system, which has been developed by the Goddard Space Flight Center. The Argon system is designed for rendezvous and proximity operations, and it is the flight cameras used during the Relative Navigation Sensor experiment in the STS-125. The vision system of the SUMO/FREND program is depicted for the mission of autonomous satellite grapple in [8]. A method by using the stereo and the 3D CAD model is given for estimating the pose in [9]. By combining the image processing method and the filtering scheme, a stereo vision based relative motion estimation method is proposed for noncooperative satellites [10]. Besides, the research based on the monocular vision is also developed by many scholars. An analysis and tests in the lab for orbital rendezvous operations are reported in [11], its sensor is the combination of a commercial web cam and two lasers. A TV-based docking control system is presented in [12] by using the monocular vision and the ISS (International Space Station) 3D model. A novel pose estimation method for noncooperative satellite is reported in [13] by recognizing solar panel triangle structure.

LIDAR sensor is another type of sensor which is commonly adopted in space relative navigation. The comprehensive review is given in [4] about the LIDAR technology as applied specifically to spacecraft relative navigation. The TriDAR system [14] has been developed by the Canadian Space Agency, which use the triangulation and scanning LIDAR technology to provide the six degrees-of-freedom pose estimation. The system was selected for the Hubble Robotic Vehicle De-orbit Module mission and tested on STS-128, STS-131, STS-135. Recently, flash LIDAR sensors [4,7,15,16,17,18,19] are developed and tested for several space program. The Ball Corp’s flash LIDAR is tested on STS-134 and is currently planned to be the primary relative navigation sensor for Orion multipurpose crew vehicle. Also, The ASC’s DragonEye flash LIDAR is selected by SpaceX for the Drogon capsule and is tested on STS-127 and STS-133. In close proximity flash LIDAR is more effective than scanning LIDAR as it can collect point cloud data at a faster frame rate. It can avoid the point cloud distortion when the target is rotating or translating. It is one of the most promising sensors for relative navigation. A method for cooperative relative navigation of spacecraft using flash LIDAR is presented in [7], for which reflectors are needed. A 3D template matching technique is designed in [15,16] for pose initial acquisition. A novel pose initialization strategy based on Oriented, Unique, and Repeatable Clustered Viewpoint Feature Histograms (OUR-CVFH) is proposed and the dual state multiplicative extended Kalman filter is combined with the pose processor to realize the relative navigation in [17]. A new method by estimating the relative pose and trajectory simultaneously using flash LIDAR is presented in [18]. Besides, the flash LIDAR can be used in other space missions, such as safe landing [19].

Several hardware-in-the-loop testbeds [20,21,22] are designed for testing the sensor performance and the algorithms for space rendezvous operations. Within the vision based navigation sensor system test campaign, hardware-in-the-loop tests on the terrestrial, robotic based facility European Proximity Operations Simulator (EPOS) 2.0 were performed to test and verify the guidance, navigation and control algorithms using real sensor measurements [20]. A hardware-in-the-loop long distance movement simulation system is designed and built at the DFKI RIC for the INVERITAS project [21]. It incorporates real hardware like mock-ups of the client and the servicer, real sensors like stereo vision, as well as sensor data processing hardware and it can simulate rendezvous and capture maneuvers. A single vision based autonomous relative navigation algorithms are presented and tested on an air-bearing table [22]. Compared with the hardware-in-the-loop systems, the advantage of the software simulation method is lower cost and easier implementation. A stereo based closed loop simulation system is designed in [23] which includes the 3D target and chaser model, the relative orbital dynamic model, and the controller model. A point cloud modeling process is described in detail in [24], and the modeling accuracy is assessed by comparing the simulated point cloud data against the test data in the laboratory experiment.

Point cloud based pose estimation methods are usually designed by registering the point cloud data collected from different viewpoints and distances. A model based method named 3D LASSO is proposed in [25] which can provide six degrees-of-freedom relative pose information by processing the 3D scanning LIDAR sensor data and is adopted in TriDAR system. A sensor different from the scanning LIDAR like the photonic mixer device (PMD) has been also used to the same goal. A spacecraft pose estimation algorithm is tested in [26] which process real-time PMD time-of-flight (ToF) camera frames to produce a six degrees-of-freedom pose estimate by 3D feature detection and feature matching. A new pose estimation method of satellites is presented in [27] by fusing PMD time-of-flight (ToF) camera and CCD sensor in order to benefit from each other sensor’s advantages, and it is tested on the European Proximity Operations Simulator (EPOS).

In this paper, the attention is focused on the pose estimation method by using the data of the flash LIDAR sensor. The same as the scanning LIDAR, the flash LIDAR sensor provides both the angle and range measurements, which can be easily converted to the three-dimensional point cloud data in the sensor frame. Unlike the previous works, in this paper, assuming that the target satellite has no cooperative markers but its model is known, a novel model based pose estimation method of satellite is designed by matching the real time 3D sensor point cloud data and the 3D model point cloud data directly. A software simulation system is devised for numerical emulation. The pose estimation method is tested with real time-of-flight sensor and satellite model on an air-bearing platform and experiment results show its effectiveness.

## 3. Proposed Pose Method

**Figure 1:**
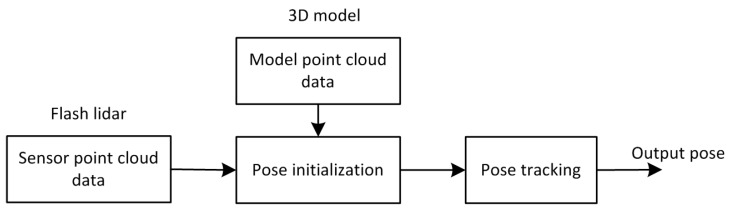
The diagram of the proposed method.

In general terms, relative pose estimation is the problem of finding the set of parameters that describe the rigid rotation and the translation between a sensor reference frame and a target reference frame. In the paper the frame translation is realized by matching the point cloud data. A brief overview of the proposed method is presented in Figure 1. When trying to follow the evolution of the relative pose of a satellite, two main steps are required: pose initialization and pose tracking. Pose initialization is performed when the first sensor point cloud data is acquired and no *a priori* information about the target relative pose is available. A novel initial method is designed based on 3D model of the satellite. Pose tracking is the subsequent step allowing the pose parameters to be updated, on the basis of the previously estimated ones, as new measurements are acquired. The details of the proposed method are given in the following part of this section.

### 3.1. Definition of Reference Frames and Pose Parameters

**Figure 2:**
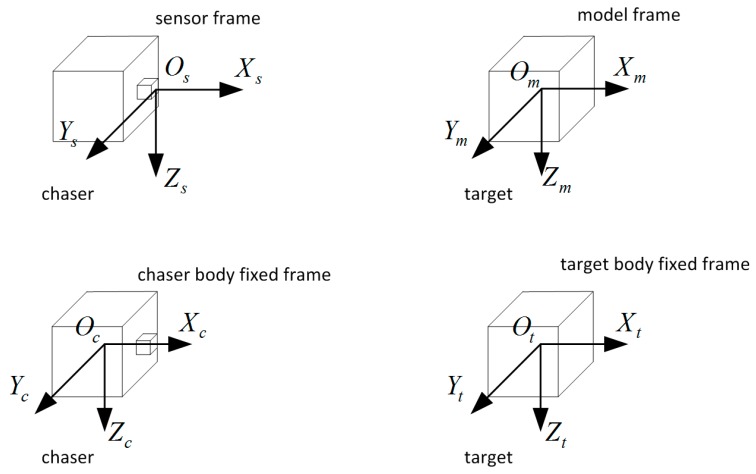
The definition of reference frames.

For the relative navigation applications of space uncooperative satellite, four reference frames are of interest: the chaser body-fixed frame, the sensor frame, the target body-fixed frame, and the target model frame, as shown in Figure 2.

The origin of the chaser body fixed frame $O_{c}-X_{c}Y_{c}Z_{c}$ and the target body fixed frame $O_{t}-X_{t}Y_{t}Z_{t}$ separately lie in the mass center of the chaser satellite and the target satellite. The orientation of the axes is determined by the pose and orbit control system. The origin of the sensor frame $O_{s}-X_{s}Y_{s}Z_{s}$ lies in the flash LIDAR sensor which is accommodated onboard the chaser. The axis $X_{s}$ increases along the optical axis away from the sensor, $Z_{s}$ is selected as parallel to a reference body axis (in the example, parallel to an edge of the spacecraft bus), and $Y_{s}$ obeys the right-hand role. The target model frame $O_{m}-X_{m}Y_{m}Z_{m}$ depends on the 3D model of the target model or the 3D point cloud data of the target. The origin of $O_{m}-X_{m}Y_{m}Z_{m}$ is defined in the centroid of the model and the orientation of the axes $X_{m},\;Y_{m},\;Z_{m}$ is parallel with the axes of the sensor frame. All reference frames may be located and oriented in a different way when required.

Assuming that the transformation matrix from $O_{s}-X_{s}Y_{s}Z_{s}$ to $O_{c}-X_{c}Y_{c}Z_{c}$ is known which is known by design, also the transformation matrix from $O_{m}-X_{m}Y_{m}Z_{m}$ to $O_{t}-X_{t}Y_{t}Z_{t}$ can be obtained offline, depending on the definition of the model frame. The pose information needed by the pose and orbit control system, which is represented by the transformation matrix from $O_{t}-X_{t}Y_{t}Z_{t}$ to $O_{c}-X_{c}Y_{c}Z_{c}$, is easily established when the transformation matrix from $O_{s}-X_{s}Y_{s}Z_{s}$ to $O_{m}-X_{m}Y_{m}Z_{m}$ can be estimated by point cloud data processing method. Thus we focus on estimating the transformation matrix from $O_{s}-X_{s}Y_{s}Z_{s}$ to $O_{m}-X_{m}Y_{m}Z_{m}$.

It is necessary to define the 6DOF relative pose parameters. The relative position is indicated as the translation vector T, as defined in Equation (1) and the relative attitude is represented as the rotation matrix R by a 312 sequence of Euler angles. Rotation about X axis by an angle φ, rotation about Y axis by an angle θ, rotation about Z axis by an angle ∅, are defined respectively as Equations (2)–(4).

(1) $$T={\left[\Delta x,\Delta y,\Delta z\right]}^{T}$$

(2) $$R_{X}(\varphi )=\left[\begin{array}{ccc} 1 & 0 & 0\\ 0 & \cos\varphi & \sin\varphi \\ 0 & -\sin\varphi & \cos\varphi \end{array}\right]$$

(3) $$R_{Y}(\theta )=\left[\begin{array}{ccc} \cos\theta & 0 & -\sin\theta \\ 0 & 1 & 0\\ \sin\theta & 0 & \cos\theta \end{array}\right]$$

(4) $$R_{Z}(\phi )=\left[\begin{array}{ccc} \cos\phi & \sin\phi & 0\\ -\sin\phi & \cos\phi & 0\\ 0 & 0 & 1 \end{array}\right]$$

(5) $$\begin{array}{l} R=R_{Y}(\theta )\times R_{X}(\varphi )\times R_{Z}(\phi )=\\ \;=\left[\begin{array}{ccc} \cos\theta \cos\phi -\sin\theta \sin\varphi \sin\phi & \cos\theta \sin\phi +\sin\theta \sin\varphi \cos\phi & -\sin\theta \cos\varphi \\ -\cos\varphi \sin\phi & \cos\varphi \cos\phi & \sin\varphi \\ \sin\theta \cos\phi +\cos\theta \sin\varphi \sin\phi & \sin\theta \sin\phi -\cos\theta \sin\varphi \cos\phi & \cos\theta \cos\varphi \end{array}\right] \end{array}$$

Considered a point, which coordinate is $P_{m}\left(x_{m},y_{m},z_{m}\right)$ in the modal frame and the corresponding matching point, which coordinate is $P_{s}\left(x_{s},y_{x},z_{s}\right)$ in the sensor frame, according to the transformation, the following Equation (6) is satisfied. Also, the transformation matrix H can be expressed by R and T as given in Equation (7).

(6) $$P_{s}=RP_{m}+T$$

(7) $$H=\left[\begin{array}{cc} R & T\\ 0 & 1 \end{array}\right]$$

### 3.2. Pose Initial Acquisition

**Figure 3:**
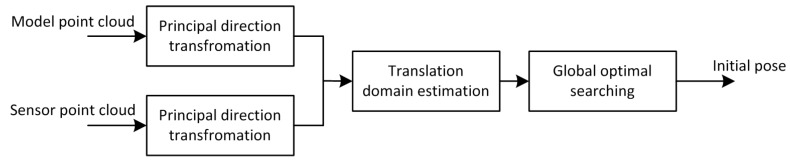
The framework of the pose initial acquisition method.

In order to solve the problem of pose initial acquisition, a novel model-based method is developed which compute the pose by directly aligning the sensor point cloud data with the prior modal point cloud data stored or built on board. In such a way, processing will not have to consider and match a number of features or to track them in a sequence of images, as in different approaches (see references [9,12,14,15,16,17,25,26,27,28,29,30]). The framework of the method is illustrated in Figure 3.

The 3D model of the target is assumed as known, which could be CAD model or 3D point cloud, due to the initial pose is uncertain between the chaser and the target, we propose a global point cloud registration algorithm to estimate the initial pose, which includes three steps, the phase of principal direction transformation, the phase of translation domain estimation, and the phase of global optimal searching.

#### 3.2.1. Principal Direction Transformation

The model point cloud data and the sensor point cloud data are defined as $P_{m}$ and $P_{s}$. The principal direction transformation is separately carried out for $P_{m}$ and $P_{s}$ and the results are $P_{m1}$ and $P_{s1}$. We adapt the $P_{s}$ to illustrate the compute procedure and generate the point cloud data $P_{s1}$.

Firstly, we compute the principal direction of the $P_{s}$ by computing the eigenvectors of the covariance matrix Cov, The matrix Cov is defined as Equation (8) and Equation (9). The eigenvectors are sorted in ascending order, which represent the XYZ axis.

(8) $$Cov=\frac{1}{n}\sum \left(p_{i}-\bar{p}\right){\left(p_{i}-\bar{p}\right)}^{T}$$

(9) $$\bar{p}=\frac{1}{n}\sum p_{i}$$

where $p_{i}$ is point of $P_{s}$, and $\bar{p}$ is the mean value, n is the number of the $P_{s}$.

The local reference frame is defined, where $\bar{p}$ is the origin and the eigenvectors are axes. Unfortunately, due to the eigenvector decomposition ambiguity, a further sign disambiguation step in the computation of the local reference frame is needed to yield a fully repeatable local reference frame. More specifically, the first eigenvector which corresponds to the minimum eigenvalue is defined as $x^{+}$, and the opposite direction is defined as $x^{-}$. Then we judge the position relation by point and point according to the Equation (10), if the point is consistent with $x^{+}$, it is added to the point collection $S_{x}^{+}$, otherwise, the point belongs to $S_{x}^{-}$. The disambiguation x axis can be established by comparing the number of point collection $S_{x}^{+}$ and $S_{x}^{-}$, so the disambiguation x axis is obtained as defined in Equation (11). The process of z axis is relevant to the maximum eigenvalue. The y axis can be obtained by cross product of z axis and  x axis. So each eigenvector is re-oriented and represented as $\left({\text{ev}}_{1},{\text{ev}}_{2},{\text{ev}}_{3}\right)$.

(10) $$\left\{ \begin{array}{l} S_{x}^{+}=\{i:(p_{i}-\bar{p})\cdot x^{+}\geq 0\}\\ S_{x}^{-}=\{i:(p_{i}-\bar{p})\cdot x^{-}\geq 0\} \end{array}\right.$$

(11) $$\text{x=}\left\{ \begin{array}{l} x^{+},\left|S_{x}^{+}\right|\geq \left|S_{x}^{-}\right|\\ x^{-},\text{otherwise} \end{array}\right.$$

Thus, we can compute $P_{s1}$ by transformation matrix $H_{s}$ as Equation (12), where $R_{s}=\left({\text{ev}}_{1},{\text{ev}}_{2},{\text{ev}}_{3}\right)$, $T_{s}=\bar{p}$. Similarly, the $P_{m1}$ is computed by transformation matrix $H_{m}$ as Equation (13).

(12) $$H_{s}=\left(\begin{array}{cc} R_{s}^{-1} & -R_{s}^{-1}T_{s}\\ 0 & 1 \end{array}\right)$$

(13) $$H_{m}=\left(\begin{array}{cc} R_{m}^{-1} & -R_{m}^{-1}T_{m}\\ 0 & 1 \end{array}\right)$$

#### 3.2.2. Translation Domain Estimation

We estimate the translation domain by using $P_{m1}$ and $P_{s1}$. Firstly, the axis aligned bounding boxes are computed separately for $P_{m1}$ and $P_{s1}$. Define $O_{m}$ as the center of the bounding box of $P_{m1}$ and $O_{s}$ as the center of the bounding box of $P_{s1}$. The origin of $P_{m1}$ and $P_{s1}$ are moved to the center of each axis aligned bounding boxes and generate new point cloud named by $P_{m2}$ and $P_{s2}$ by transformation matrix $H_{O_{m}}$ and $H_{O_{s}}$ as Equations (14) and (15).

(14) $$H_{O_{m}}=\left(\begin{array}{cc} I & -O_{m}\\ 0 & 1 \end{array}\right)$$

(15) $$H_{O_{s}}=\left(\begin{array}{cc} I & -O_{s}\\ 0 & 1 \end{array}\right)$$

where I is 3×3 unit matrix.

Define the length of XYZ axis of the bounding box of $P_{m1}$ as $l_{\text{mx}}$, $l_{\text{my}}$, $l_{\text{mz}}$. The corresponding length of $P_{s1}$ as $l_{\text{sx}}$, $l_{\text{sy}}$, $l_{\text{sz}}$. So we can compute the translation domain by Equation (16).

(16) $$\left\{ \begin{array}{l} x_{t}=\left\{x:-(\left|l_{mx}-l_{sx}\right|/2+x_{\Delta })\leq x\leq (\left|l_{mx}-l_{sx}\right|/2+x_{\Delta })\right\}\\ y_{t}=\left\{y:-(\left|l_{my}-l_{sy}\right|/2+y_{\Delta })\leq y\leq (\left|l_{my}-l_{sy}\right|/2+y_{\Delta })\right\}\\ z_{t}=\left\{z:-(\left|l_{mz}-l_{sz}\right|/2+z_{\Delta })\leq z\leq (\left|l_{mz}-l_{sz}\right|/2+z_{\Delta })\right\} \end{array}\right.$$

where $x_{t},\;y_{t},\;z_{t}$ represent the translation range of XYZ axis. $x_{\Delta },\;y_{\Delta },\;z_{\Delta }$ represent the compensation factor. Due to the axis aligned bounding box is not the minimum bounding box, the compensation factor α is designed and its value is set to an empirical value such as 0.05 in Equation (17).

(17) $$\left\{ \begin{array}{l} x_{\Delta }=\alpha \times l_{mx}\\ y_{\Delta }=\alpha \times l_{my}\\ z_{\Delta }=\alpha \times l_{mz} \end{array}\right.$$

#### 3.2.3. Global Optimal Searching

A global optimal searching method is used to matching the $P_{m2}$ and $P_{s2}$. The branch-and-bound (BnB) is combined with the Iterative Closest Point (ICP) algorithm to search the 3D space efficiently [31]. In this paper, the angle-axis representation is used, the entire space formed by XYZ axes rotations can be compactly represented as a solid radius –π ball in 3D space. So we set the rotation domain as ${\left[–\pi ,\pi \right]}^{3}$ that encloses the π ball. For the translation part we set the translation domain as illustrate in Equation (16).

The searching process is the same as that in [31] and it is summarized as follows. Use the BnB to search the space, whenever a better solution is found, call ICP to refine the objective function value. Use ICPs result as an updated upper bound to continue the above BnB search until convergence. During BnB searches, the octree data structure is used and the process is repeated.

We define the matrix $H_{g}$ as the global searching result. So we can get the initial pose matrix $H_{f}$ by Equation (18).

(18) $$H_{f}={\left(H_{O_{m}}H_{m}\right)}^{-1}H_{g}H_{O_{s}}H_{s}$$

We can get the 6DOF relative pose parameters from the $H_{f}$ which represents the transformation matrix from $O_{s}-X_{s}Y_{s}Z_{s}$ to $O_{m}-X_{m}Y_{m}Z_{m}$.

### 3.3. Pose Tracking

After the initial pose is known, we can execute the pose tracking process to generate the continuous pose output by using the sensor point cloud data at a frame rate.

If the previous pose of the satellite is known, the problem of estimating the current pose can be simplified by restricting the search to solutions that are close the previous pose. In this paper, an Iterative Closest Point (ICP) algorithm can be used for this task to align the current sensor point cloud data with the model point cloud data.

Assuming that the previous transformation matrix is defined as $H_{k-1}$, and the current sensor point cloud data is $P_{s_{k}}$, the process is depicted as follows.

Firstly, we transform the $P_{s_{k}}$ by the matrix $H_{k-1}$, then the converted sensor point cloud date is aligned with the model point cloud data $P_{m}$ by using the ICP algorithm and the current transformation matrix $H_{k}$ is obtained. Also, the 6DOF relative pose parameters is obtained from the $H_{k}$.

Specifically, in this work, the ICP error is the mean squared distance of the corresponding points between the two point clouds. The ICP algorithm is stopped as soon as the variation of the ICP error among two subsequent iterations becomes less than 10^−6^ m^2^. Moreover, a maximum number of 20 iterations is set to prevent the ICP algorithm from taking too long.

## 4. Simulation System

The simulation system allows extensive pose estimation performance simulations prior to field testing, saving development cost and providing performance metrics of the pose estimation algorithm. It provides a true pose of the test objects and the simulated sensor point cloud data simultaneously so that the pose estimation error can be quantified.

### 4.1. Target Model and Sensor Parameters

We focus on how to generate the sensor data, the target model and sensor parameters are needed.

The model point cloud data can be obtained offline though the 3D CAD model of the target by finite element analysis software; or can be obtained online by the technology of three-dimensional reconstruction through the chaser flying around the target. In this paper, the 3D CAD model of the target is used to generate the model point cloud data by means of the UG finite element analysis software. The size of mesh grids is set to 10mm, which is also considered as the spatial resolution of the model point cloud data. The model point cloud data is dense and also adopted in the experiments.

The parameters of the sensor include focus length f, pixel resolution h×v, pixel size $d_{x}\times d_{y}$, field of view $\alpha_{h}\times \alpha_{v}$, which usually are fixed to a certain type of sensor and given in the product datasheet.

### 4.2. Generate Simulated Point Cloud Data

**Figure 4:**
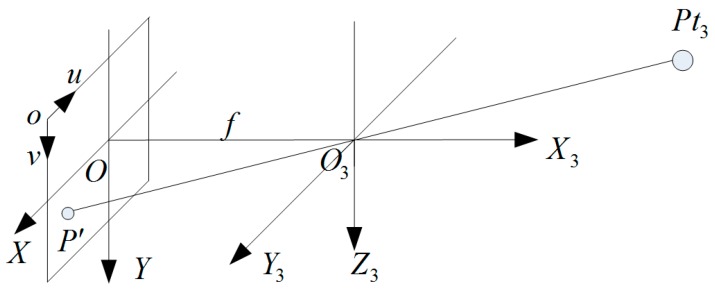
The imaging mapping graph.

We aim to generate simulated point cloud data by using the target model point cloud data. Suppose a point $P_{m}\left(x_{m},y_{m},z_{m}\right)$, whose coordinates are known in the model frame, we aim to compute the measured point $P_{s}\left(x_{s},y_{x},z_{s}\right)$. For each point in the model, the same process steps are executed as follows:

1. Set the transformation matrix $H_{t}$ from the model frame to the sensor frame, which can be computed by the known observed position, we get the corresponding point $P_{t1}\left(x_{1},y_{1},z_{1}\right)$ in the sensor frame.
  By setting the rotation angle (φ,θ,∅), and the observed position (x,y,z), the matrix $H_{t}$ can be obtained with the Equations (1)–(5). Also the setting parameters and matrix $H_{t}$ represent the true pose value.
2. For $P_{t1}\left(x_{1},y_{1},z_{1}\right)$, we judge whether the point lies in the field of view of the sensor which is computed according to the sensor parameters by Equation (19).
  (19) $$\{\begin{array}{c} H_{range}=\left[-D\tan\left(\alpha_{h}/2\right),D\tan\left(\alpha_{h}/2\right)\right]\\ V_{range}=\left[-D\tan\left(\alpha_{v}/2\right),D\tan\left(\alpha_{v}/2\right)\right] \end{array}$$
  where D is the distance of optical axis, its value is $x_{1}$ in the paper. $\alpha_{h}$ and $\alpha_{v}$ are the horizontal and vertical view angle. $H_{range}$ and $V_{range}$ are the horizontal and vertical observable range. If $y_{1}$ belongs to $H_{range}$ and $z_{1}$ belongs to $V_{range}$, the point is reserved to $P_{t2}\left(x_{2},y_{2},z_{2}\right)$, otherwise it is discarded.
3. For each point $P_{t2}\left(x_{2},y_{2},z_{2}\right)$ of the point cloud, considering the measurement distance error always exists, we firstly add an random distance value Δd as Equation (20) and get the point $P_{t3}\left(x_{3},y_{3},z_{3}\right)$.
  (20) $${\left\|P_{t2}\right\|}^{2}+\Delta d={\left\|P_{t3}\right\|}^{2}$$
  where Δd is set to a random value in the range $\left[-\Delta d_{\max},\Delta d_{\max}\right]$. $\Delta d_{\max}$ is the maximum absolute error and can be given in the dataset of the sensor.
4. For each point $P_{t3}\left(x_{3},y_{3},z_{3}\right),$ we compute its pixel coordinates as given in the Equations (21) and (22) and Figure 4. If multiple points have the same pixel coordinates $P^{\prime }\left(u,v\right)$, we simply choose the point, which the distance $x_{3}$ is minimum, as the final point $P_{s}\left(x_{s},y_{s},z_{s}\right)$.
  (21) $$\frac{x_{3}}{f}=\frac{z_{3}}{y};\frac{x_{3}}{f}=\frac{y_{3}}{x}$$
  (22) $$\left\{ \begin{array}{l} u=-x/d_{x}+u_{0}\\ v=y/d_{y}+v_{0} \end{array}\right.$$
  where f is the focus length, (x,y) is the corresponding coordinate in the O−XY frame, and (u,v) is the pixel coordinate in the o−uv frame. $\left(u_{0},v_{0}\right)$ is the center point of the image plane. $d_{x}$ and $d_{y}$ are the horizontal and vertical pixel size.

**Figure 5:**
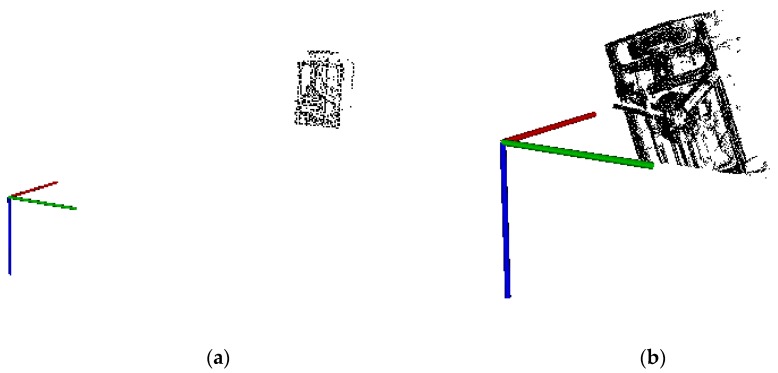
An example of the simulated point cloud data (the sensor locates at different view position, (**a**) the distance is 10 m; (**b**) the distance is 2 m).

An example of sensor point cloud data generated by the simulator is shown in 3D view in Figure 5. The output of the time-of-flight sensor is comparable to the one of a flash LIDAR, so the time-of-flight sensor can be used as a replacement for the inexpensive lab testing. The SR4000 of MESA company is a typical time-of-flight sensor [32] and its parameters are presented in [33]. The sensor SR4000 is also adopted for numerical simulation experiments and field experiments in this paper.

In Figure 5, the axis represents the view position of the sensor, the data in left is sparse due to the far distance, and the data in right is dense due to the close distance, also the pose is different. We can see that the simulation can generate the senor data, and provide the truth value of the pose simultaneously. In Figure 5, the number of points in the model point cloud is about 80,000; the number of points in the simulated point cloud in 10 m distance is about 700, and the number of points in the simulated point cloud in 2 m distance is about 5000.

## 5. Experiments and Discussion

### 5.1. Test Setup

In order to test the algorithm, we have performed two experiments, including numerical simulation experiments, which use the point cloud data built as depicted in Section 4, and a field experiment, which use the sensor SR4000 and the mechanical models. 

The pose algorithm and the simulation system are implemented with C++ codes, the detailed settings and results are given in Section 5.2.

The field experiment, carried on with a SR4000 sensor and the actual mechanical models for the target and the chaser, is discussed in Section 5.3.

### 5.2. Numerical Simulation Experiments

Several numerical simulation experiments are conducted with different simulation conditions. These are given in the following part. Experiments 1 and 2 are tested for pose initial acquisition; Experiments 3 and 4 are tested for relative motion with different motion conditions.

Suppose the sensor is SR4000, $\Delta d_{\max}$ is set to 1cm and the model point cloud data is known, we generate the simulated point cloud data for each experiment by using the model point cloud data and the corresponding simulation conditions, as given in Section 4. Meanwhile, the true pose values can be obtained so the pose error curves are given in every experiment. 

#### 5.2.1. Experiment 1

To test the initial pose acquisition method, a simulated experiment is carried out under the following conditions.

**Figure 6:**
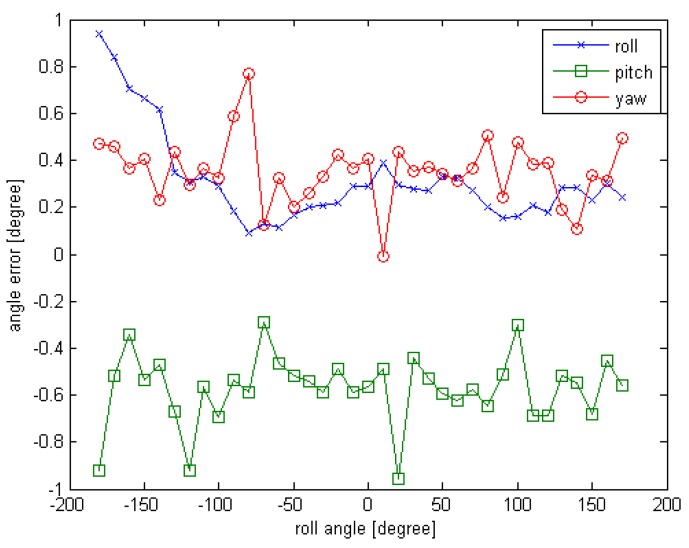
The rotation error curve of Experiment 1 for initial pose.

**Figure 7:**
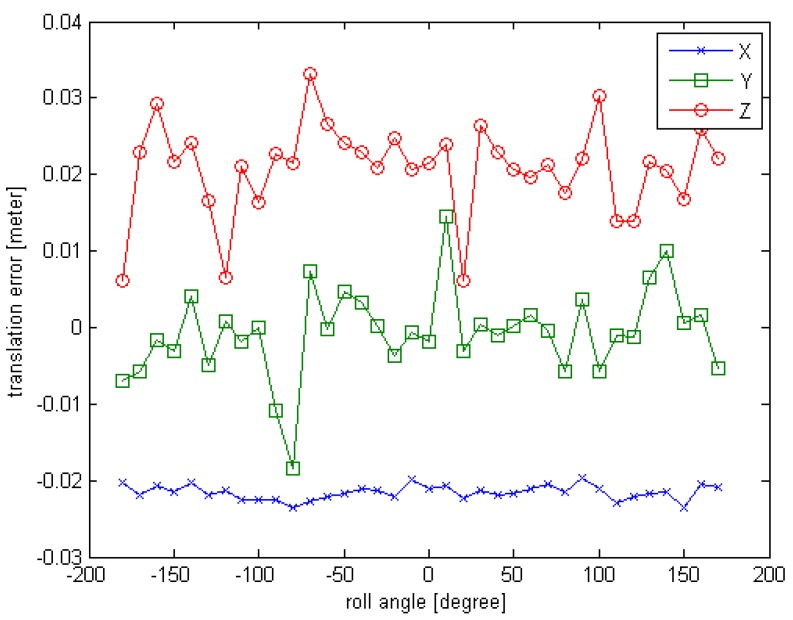
The translation error curve of Experiment 1 for initial pose.

The observed position (x,y,z) is set to (10,0,0), the units are in meters; the initial rotation angle (φ,θ,∅) is set to (−180,0,0), the units are in degrees. For each simulated point cloud data, the φ is changing from −180° to 180° at 10° interval. So a series of simulated point cloud data can be obtained and the truth pose is known, the computed result of the proposed initial pose acquisition method is obtained as depicted in Section 3.2. The error curves are given in Figure 6 and Figure 7.

From the Figure 6 and Figure 7, we can see that the initial pose acquisition method can cope with an arbitrary viewing point. The rotation error is about 1° and the translation error is less than 4 cm.

#### 5.2.2. Experiment 2

**Figure 8:**
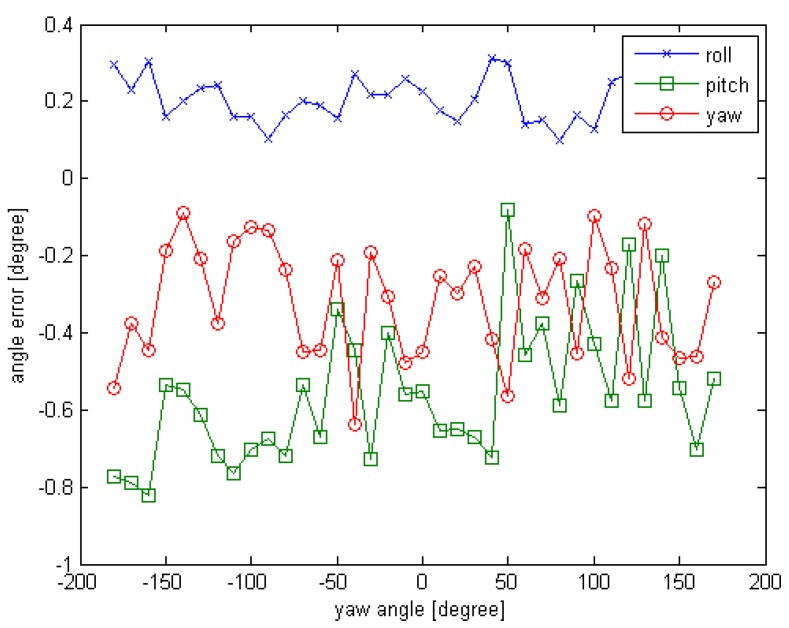
The rotation error curve of Experiment 2 for initial pose.

**Figure 9:**
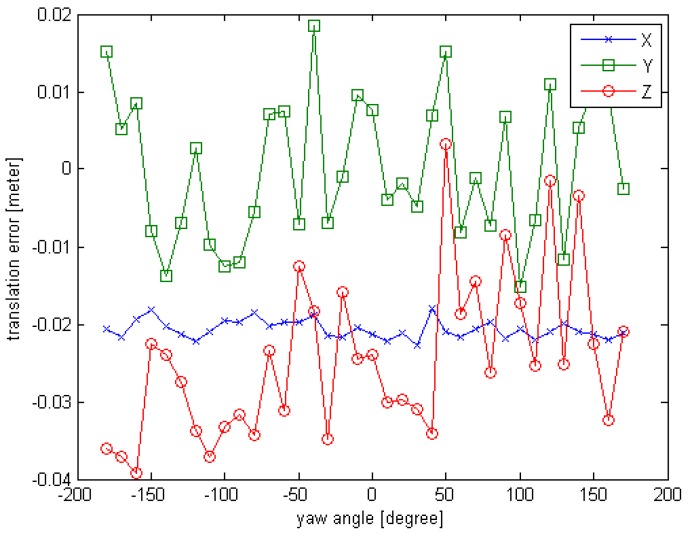
The translation error curve of Experiment 2 for initial pose.

Another experiment is conducted to test the effectiveness of the initial pose acquisition method. The simulation condition is as follows. The observed position (x,y,z) is set to (10,0,0); the initial rotation angle (φ,θ,∅) is set to (45,45,−180). For each simulated point cloud data, the ∅ is changing from −180° to 180° at 10° interval. In this test, different from Experiment 1, the roll angle and the pitch angle are set to a non-zero fixed value, and the yaw angle changes in each sensor data. The error curves are given in Figure 8 and Figure 9.

From the Figure 8 and Figure 9, we can see that the initial pose acquisition method can gain the initial pose accurately. The rotation error is about 1° and the translation error is less than 4 cm.

#### 5.2.3. Experiment 3

**Figure 10:**
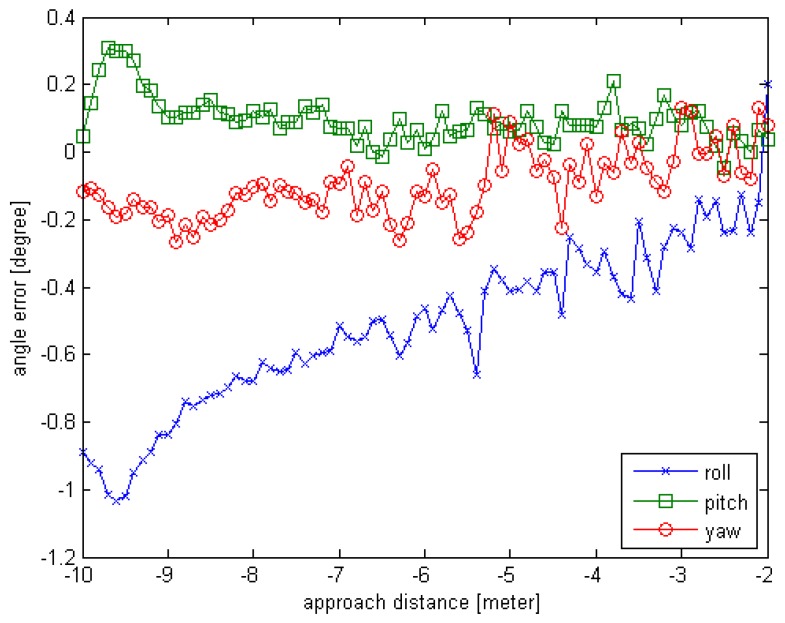
The rotation error curve of Experiment 3 for relative motion.

**Figure 11:**
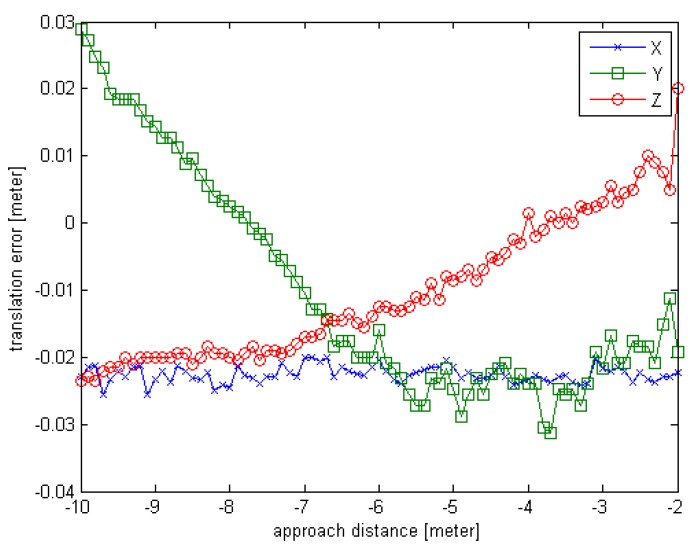
The translation error curve of Experiment 3 for relative motion.

To test the proposed method including the pose initial acquisition and the pose tracking, simulated experiment is carried out under the following conditions. Suppose the chaser is in rendezvous motion with uniform velocity to the target, and the target is rotating on X-axis with a constant angular velocity, we generate the simulated point cloud data as given in Section 4. The observed position (x,y,z) is set to (10,0,0), the units are in meters; the initial rotation angle (φ,θ,∅) is set to (0,0,0), the units are in degrees. For each simulated point cloud data, the φ is changing from 0° to 160° at 2° interval and the x distance is changing from 10 m to 2 m at 0.1 m interval. So the number of the simulated sensor point cloud data is 81. Also the truth pose values can be obtained while the simulated sensor data are generated. The estimated pose error is computed and the error curves are given in Figure 10 and Figure 11. The negative sign about approach distance indicates that the chaser is moving towards the target along the X-axis.

From the Figure 10 and Figure 11, we can see that the proposed relative pose estimation method based on point cloud can compute the real-time pose of a target even if the target is fast. The rotation error is less than 1° and the translation error is about 3 cm. Also, we can see that the errors in close distance are smaller than the errors in far distance because as the chaser approaches the target, the sensor can obtain more points and generate more accurate estimation results.

#### 5.2.4. Experiment 4

**Figure 12:**
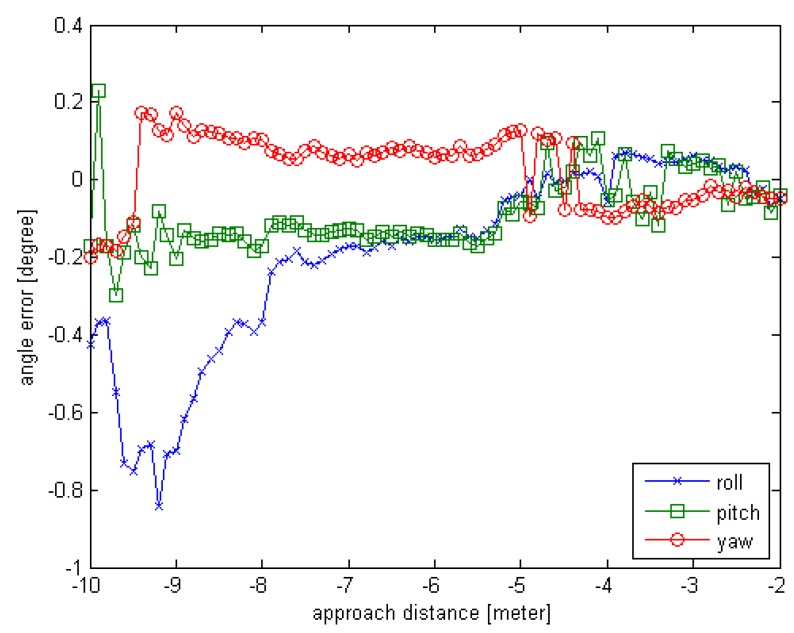
The rotation error curve of Experiment 4 for relative motion.

**Figure 13:**
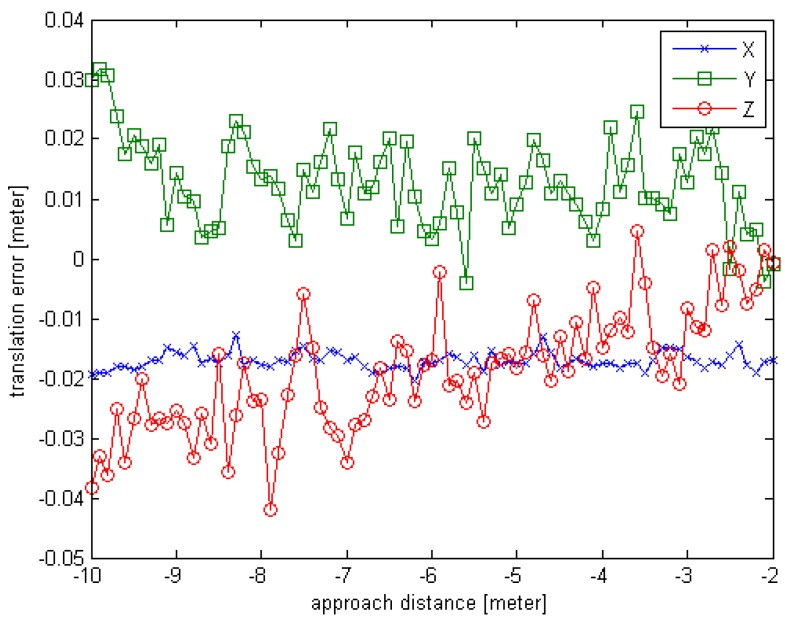
The translation error curve of Experiment 4 for relative motion.

Another experiment is conducted to test the proposed method including the pose initial acquisition and the pose tracking. The simulation condition is as follows. Suppose the chaser is in rendezvous motion with uniform velocity to the target, and the target is rotating on X-axis and Z-axis simultaneously. The observed position (x,y,z) is set to (10,0,0); the initial rotation angle (φ,θ,∅) is set to (0,45,0). For each simulated point cloud data, the φ is changing from 0° to 80° at 1° interval; the ∅ is also changing from 0° to 80° at 1° interval; the x distance is changing from 10 m to 2 m at 0.1 m interval. So the number of the simulated sensor data is 81. In this test, different from Experiment 3, rotation exists on the roll and the yaw in the adjacent sensor data and the pitch angle is set to a non-zero fixed value. The error curves are given in Figure 12 and Figure 13.

From the Figure 12 and Figure 13, we can see that the proposed relative pose estimation method can estimate the real-time relative pose effectively. The rotation error is less than 1° and the translation error is less than 4 cm. Also, we can see that the errors decrease as the approach distance decreases.

### 5.3. Field Experiments

In order to test the real performance of the algorithm, a field experiment is carried out. 

The hardware testing setup consists of mechanical models, servo control systems. Two mechanical models are adapted, one with the time-of-flight sensor as the chaser and the other one as the target. The servo control systems control the motion of the 6DOF mechanical model. A detailed explanation of the hardware is beyond this article’s scope. The SR4000 is used to obtain the point data. It illuminates the entire field of view of 43° by 34° and collects the point cloud data simultaneously using a 176 × 144 CCD detector array. The maximum range is 10m with measurement accuracy of 1 cm.

The target is still, and the real-time pose estimation results are used to drive the chaser. Due to the limitation of the experimental site and equipment, the working distance is set from 4 m to 0.5 m. The real-time rotation angle and translation value are given in Figure 10 and Figure 11.

Although the simulation results seem good as shown in Section 5.2, however the accuracy of the pose estimation may be affected seriously by many factors in ground experiment. The factors include the material and structure of the target, the installation position of the sensor, the characteristics of the sensor, and the performance of the servo system and so on.

**Figure 14:**
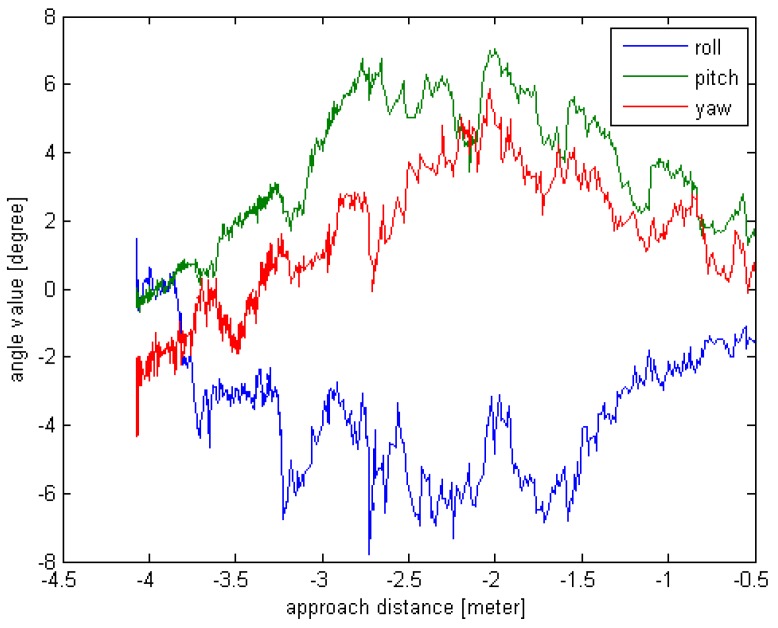
The rotation angle value curve for field experiment.

**Figure 15:**
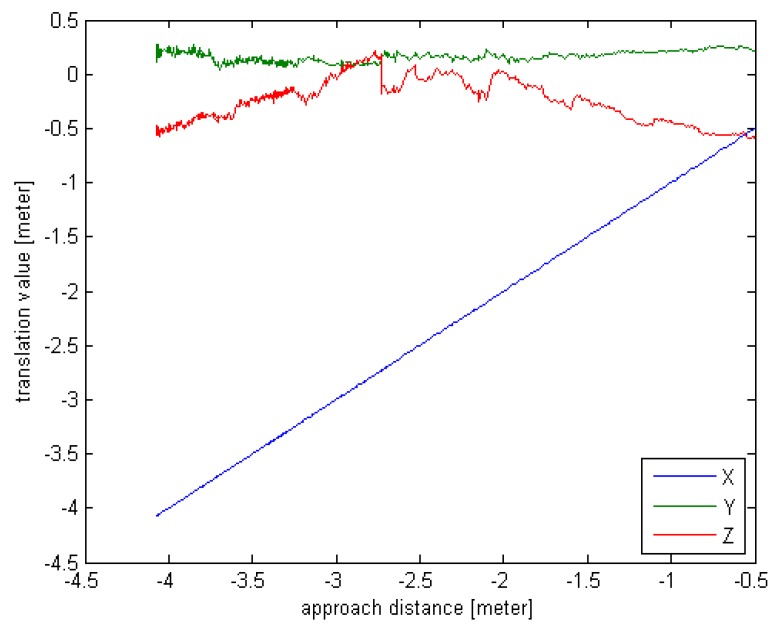
The translation value curve for field experiment.

From the Figure 14 and Figure 15, we can see that the chaser can move toward the target by utilizing the real-time pose estimation results. Since no truth value is provided, the measurement errors are absent. It is obvious that the rotation angle value and the Z-axis translation value vary significantly when the approach distance is from 3 m to 2 m. The origin of this effect may be related to multiple path reflections. The other pose results are reliable and the actual motion trajectory of the chaser meets the requirements.

### 5.4. Discussion

The numerical simulation experiments and results are presented in Section 5.2. From Experiments 1 and 2, we can see that the proposed method can effectively estimate the initial pose. The reason is that the translation domain is computed effectively and the global optimal searching is adopted, which can get the best matching result for arbitrary initial pose by using the distribution characteristics of the point clouds. As presented in the Experiments 3 and 4, the real-time relative pose can be obtained while the motion exists between the chaser and the target. The reason is the ICP algorithm is efficient and is initialized by the previous pose to speed the convergence. Besides, we can see that the smaller the rotation angle in adjacent sensor data, the smaller the errors. It is obvious that the results are more accurate when the pose changes slightly than the results given when the pose changes rapidly.

However, in the field experiments, the accuracy not only depends on the pose estimation method but may also be affected by many factors such as the performance parameters of the sensor, the noise of point cloud in real scenes, and so on. A pre-processing of the sensor point cloud data is needed which aims to separate the target from complicated backgrounds in the field experiments. The confidence map of the output of the sensor is used, which represents a measure of probability or confidence of how the distance measurement is expected to be. Low confidence represents that the corresponding point is unreliable, so it is easy to obtain the target by a preset confidence threshold.

We evaluate the runtime of the proposed method by computing the average processing time. The CPU is dual core 2.9 GHz and the RAM is 3 GB. The code is implemented based on the Point Cloud Library (PCL) which is a third party library for 2D/3D image and point cloud processing [34]. The total average execution time is about 50 ms which corresponds to roughly 20 FPS.

## 6. Conclusions

A relative pose estimation method of satellite in close range is proposed, which uses the known target model and the point cloud data generated by the flash LIDAR sensor. The method estimates the relative pose directly on the basis of the dense point cloud data and can deal with large initial pose difference and rapid pose changes effectively. There is no need for the cooperative markers on the target satellite and the process of feature detection and feature tracking. The simulation system is designed to generate the simulated sensor point cloud data and truth pose value simultaneously by the various motion conditions. So it allows extensive pose estimation performance simulations for the pose estimation method and tests the performance of the specific sensor prior to field testing, saving cost and providing performance metrics of pose estimation algorithms under evaluation. The numerical simulated experiment results denote that the proposed pose estimation method is accurate and efficient. Also, the field experiment with the hardware system was conducted in order to test the performance on the ground.

The flash LIDAR sensor is a promising technology in space applications due to its unique combination of advantages (low power, high framerate, low mass, robustness). It can provide an alternative method for future relative navigation tasks in close ranges. Regarding future research, some improvements will be thought of on these aspects: (1) The point cloud filtering method will be designed and adopted to reduce the influence of noise and artifacts in field experiments; (2) Other high performance sensors will be modeled and tested by using the proposed pose estimation method and the simulation system.

## References

[1] Bao W.M. (2015). Present situation and development tendency of aerospace control techniques. Acta Autom. Sinica.

[2] Flores-Abad A., Ma O., Pham M., Ulrich S. (2014). A review of space robotics technologies for on-orbit servicing. Prog. Aerosp. Sci..

[3] Rebordao J.M. Space optical navigation techniques: An overview. Proceedings of the SPIE 8th Iberoamerican Optics Meeting and 11th Latin American Meeting on Optics, Lasers, and Applications.

[4] Christian J.A., Cryan S. A survey of LIDAR technology and its use in spacecraft relative navigation. Proceedings of the AIAA Guidance, Navigation, and Control Conference.

[5] Wei X.Q., Huang J.M., Chen F., Zhang X.G., Jin Y.Q., Xing G.Q. (2012). A study on autonomous video navigation in close range with a cooperative target. Manned Spacefl..

[6] Galante J.M., Van Eepoel J., Strube M., Gill N., Gonzalez M., Hyslop A., Patrick B. Pose measurement performance of the argon relative navigation sensor suite in simulated flight conditions. Proceedings of the AIAA Guidance, Navigation, and Control Conference.

[7] Christian J.A., Robinson S.B., D’Souza C.N., Ruiz J.P. (2014). Cooperative relative navigation of spacecraft using flash light detection and ranging sensors. J. Guidance Control Dyn..

[8] Obermark G., Creamer G., Kelm B.E., Wagner W., Henshaw C.G. SUMO/FREND vision system for autonomous satellite grapple. Proceedings of the SPIE Sensors and Systems for Space Applications.

[9] Terui F., Kamonura S., Nishida S. Motion estimation to a failed satellite on orbit using stereo vision and 3D model matching. Proceedings of the 9th International Conference on Control, Automation, Robotics and Vision.

[10] Segal S., Carmi A., Gurfil P. (2014). Stereo vision-based estimation of relative dynamics between noncooperative satellites theory and experiments. IEEE Trans. Control Syst. Technol..

[11] Palmerini G., Sabatini M., Gasbarri P. Analysis and tests of visual based techniques for orbital rendezvous operations. Proceedings of the IEEE Aerospace Conference.

[12] Stepanov D., Bakhshiev A., Gromoshinskii D., Kirpan N., Gundelakh F. (2015). Determination of the relative position of space vehicles by detection and tracking of natural visual features with the existing TV-cameras. Commun. Comput. Inf. Sci..

[13] Song J.Z., Cao C.X. (2015). Pose self-measurement of noncooperative spacecraft based on solar panel triangle structure. J. Robot..

[14] English C., Okouneva G., Saint-Cyr P., Choudhuri A., Luu T. (2011). Real-time dynamic pose estimation systems in space lessons learned for system design and performance evaluation. Int. J. Intell. Control. Syst..

[15] Opromolla R., Fasano G., Rufino G., Grassi M. (2015). Uncooperative pose estimation with a LIDAR-based system. Acta Astronaut..

[16] Opromolla R., Fasano G., Rufino G., Grassi M. (2015). A model-based 3D template matching technique for pose acquisition of an uncooperative space object. Sensors.

[17] Woods J.O., Christian J.A. (2016). LIDAR-based relative navigation with respect to non-cooperative objects. Acta Astronaut..

[18] McMahon J.W., Gehly S., Axelrad P. Enhancing relative attitude and trajectory estimation for autonomous rendezvous using Flash LIDAR. Proceedings of the AIAA/AAS Astrodynamics Specialist Conference.

[19] Amzajerdian F., Roback V.E., Bulyshev A.E., Brewster P.F. Imaging flash LIDAR for safe landing on solar system bodies and spacecraft rendezvous and docking. Proceedings of the SPIE Laser Radar Technology and Applications XX; and Atmospheric Propagation XII.

[20] Benninghoff H., Rems F., Boge T. (2014). Development and hardware-in-the-loop test of a guidance navigation and control system for on-orbit servicing. Acta Astronaut..

[21] Paul J., Dettmann A., Girault B., Hilljegerdes J., Kirchner F., Ahrns I., Sommer J. (2015). INVERITAS a facility for hardware-in-the-loop long distance movement simulation for rendezvous and capture of satellites and other autonomous objects. Acta Astronaut..

[22] Sabatini M., Palmerini G.B., Gasbarri P. (2015). A testbed for visual based navigation and control during space rendezvous operations. Acta. Astronaut..

[23] Xu W.F., Liang B., Li C., Liu Y., Qiang W.Y. (2009). The approach and simulation study of the relative pose measurement between spacecrafts base on stereo vision. J. Astronaut..

[24] Lim T.W. (2015). Point cloud modeling using the homogeneous transformation for non-cooperative pose estimation. Acta Astronaut..

[25] Ruel S., Luu T., Anctil M., Gagnon S. Target localization from 3D data for on-orbit autonomous rendezvous and docking. Proceedings of the IEEE Aerospace Conference.

[26] Tzschichholz T., Ma L., Schilling K. (2011). Model-based spacecraft pose estimation and motion prediction using a photonic mixer device camera. Acta. Astronaut..

[27] Tzschichholz T., Boge T., Schilling K. (2015). Relative pose estimation of satellites using PMD-/CCD-sensor data fusion. Acta. Astronaut..

[28] Sharma S., D’Amico S. (2016). Comparative assessment of techniques for initial pose estimation using monocular vision. Acta. Astronaut..

[29] Gasbarri P., Sabatini M., Palmerini G.B. (2014). Ground tests for vision based determination and control of formation flying spacecraft trajectories. Acta. Astronaut..

[30] Chien C.H., Baker K. Pose estimation for servicing of orbital replacement units in a cluttered environment. Proceedings of the IEEE International Conference on Robotics and Automation.

[31] Yang J., Li H., Jia Y. Go-ICP: Solving 3D Registration Efficiently and Globally Optimally. Proceedings of the IEEE International Conference on Computer Vision.

[32] Piatti D., Rinaudo F. (2012). SR-4000 and CamCube3.0 Time of Flight (ToF) Cameras: Tests and comparison. Remote Sens..

[33] SR4000 Datasheet. http//www.mesa-imaging.ch.

[34] Point Cloud Library. http//www.pointclouds.org.

